# Efficient computation of contributional diversity metrics from microbiome data with *FuncDiv*

**DOI:** 10.1093/bioinformatics/btac809

**Published:** 2022-12-15

**Authors:** Gavin M Douglas, Sunu Kim, Morgan G I Langille, B Jesse Shapiro

**Affiliations:** Genome Centre, McGill University, Montréal, QC H3A 0G1, Canada; Department of Microbiology & Immunology, McGill University, Montréal, QC H3A 2B4, Canada; Department of Microbiology & Immunology, McGill University, Montréal, QC H3A 2B4, Canada; Department of Pharmacology, Dalhousie University, Halifax, NS B3H 4R2, Canada; Genome Centre, McGill University, Montréal, QC H3A 0G1, Canada; Department of Microbiology & Immunology, McGill University, Montréal, QC H3A 2B4, Canada

## Abstract

**Motivation:**

Microbiome datasets with taxa linked to the functions (e.g. genes) they encode are becoming more common as metagenomics sequencing approaches improve. However, these data are challenging to analyze due to their complexity. Summary metrics, such as the alpha and beta diversity of taxa contributing to each function (i.e. contributional diversity), represent one approach to investigate these data, but currently there are no straightforward methods for doing so.

**Results:**

We addressed this gap by developing *FuncDiv*, which efficiently performs these computations. Contributional diversity metrics can provide novel insights that would be impossible to identify without jointly considering taxa and functions.

**Availability and implementation:**

*FuncDiv* is distributed under a GNU Affero General Public License v3.0 and is available at https://github.com/gavinmdouglas/FuncDiv.

**Supplementary information:**

Supplementary data are available at *Bioinformatics* online.

## 1 Introduction

Metagenome-assembled genomes (MAGs) are now a common data type generated from metagenomics sequencing data. MAG-based analyses typically focus on two data types: (i) the relative abundances of MAGs over multiple samples and (ii) the gene families encoded by each MAG. In other words, the abundances of microbial genes (and higher-level functions) across different samples can be partitioned into the contributions of different organisms when MAGs are used. This contrasts with ‘bag-of-genes’ approaches, where function relative abundances are inferred from raw reads or non-binned contigs. MAG-based taxon-function information allows the key taxa that drive functional differences to be identified, which is more biologically informative than typical analyses based on function-relative abundances alone (Bradley *et al.*, 2018; Manor and Borenstein, 2017).

Linked taxa-function data provide insight into the diversity of taxa that encode a specific function (i.e. the taxonomic contributors) (Douglas and Langille, 2021; Franzosa *et al.*, 2018). This diversity can be assessed within samples (alpha) or between samples (beta) and is commonly computed based on the overall taxonomic relative abundances in microbiome datasets. The complication with linked taxon-function data (which is referred to as ‘contributional data’) is that an additional set of computations is required for each separate function, which greatly increases the computational burden. There is also no existing workflow that enables researchers to compute diversity metrics for contributional data specifically (which we refer to as ‘contributional diversity’). Although diversity metrics for individual taxon abundance tables can be computed with tools like *vegan* and *QIIME 2* (Bolyen *et al.*, 2019), it would require bioinformatics expertise to efficiently use such approaches in a custom contributional diversity workflow. This is an issue, as many microbiome researchers lack the programming background to develop their own bioinformatics methods.

To address these challenges, we developed the *FuncDiv* R package, which provides straightforward workflows for researchers to compute both alpha and beta contributional diversity metrics. This program enables researchers to investigate the contributional diversity of taxa-function-linked microbiome data, which can provide novel insight into microbial communities.

## 2 Materials and methods

### 2.1 Overview

**Fig. 1.**
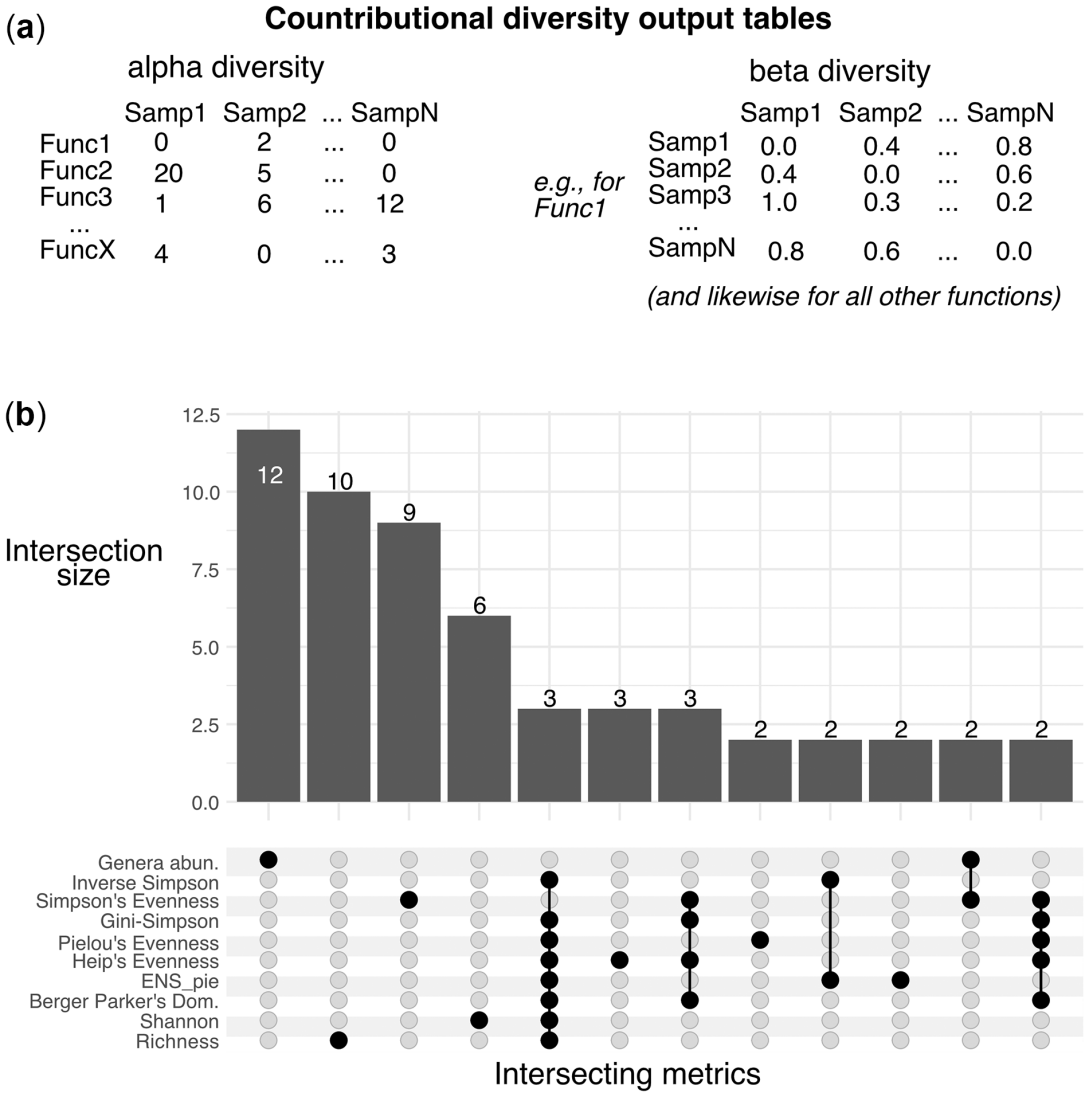
(**a**) Examples of contributional diversity output tables, indicating the breakdown by different function and sample combinations. (**b**) Upset plot of the intersecting MetaCyc pathways in the top 20 most informative features in each respective example random forest classification model based on each data type. ‘Genera abun.’ corresponds to the summed and transformed relative abundance of genera that encode each pathway per sample. All other categories are alpha diversity metrics computed with *FuncDiv*

*FuncDiv* computes the contributional diversity for each function in a dataset (Fig. 1a). Functions can correspond to any arbitrary category. For instance, KEGG orthologs (Kanehisa *et al.*, 2016), eggNOG orthogroups (Huerta-Cepas, 2019) and MetaCyc pathways (Caspi *et al.*, 2016) would all be valid function types. Entirely different data types, such as taxa-linked metabolome data, could also be input. Contributional alpha diversity corresponds to the diversity of taxa (usually based on their relative abundances) that encode a given function in a sample. Contributional beta diversity corresponds to the divergence in the presence and/or relative abundances of taxa that encode a given function between different samples.

### 2.2 Input data

The primary input tables to *FuncDiv* are (i) a table of taxonomic (e.g. MAG) relative abundances across samples and (ii) a table containing the function (e.g. gene) copy numbers found within each taxon. For the purposes of *FuncDiv*, single-copy and multi-copy functions per taxon are treated identically (i.e. taxa are classified as either contributors or non-contributors of a function). Users can also input a combined table of taxa and functions, which we refer to as contributional format. This format is output by several microbiome-based tools, such as *HUMAnN3* (Beghini *et al.*, 2021) and *PICRUSt2* (Douglas *et al.*, 2020). *FuncDiv* provides utility functions for converting between these table formats.

### 2.3 Alpha diversity

The key function for computing contributional alpha diversity is *alpha_div_contrib*. Fifteen alpha diversity metrics are currently implemented, such as Faith’s phylogenetic diversity (Faith, 1992) and the Gini-Simpson index. Most of these metrics are R re-implementations of alpha diversity metrics from the *scikit-bio* Python package. Custom metrics (as defined by R functions) can also be specified by users. A key step in the alpha diversity workflow is to format the input data into a large list object that is easily parsed in R with multiple cores running in parallel. This step is performed using custom C++ code integrated with the *Rcpp* (Eddelbuettel and Francois, 2011) and *RcppArmadillo* (Eddelbuettel and Sanderson, 2014) R packages.

### 2.4 Beta diversity

Contributional beta diversity is computed with the *beta_div_contrib* function. In this case, beta diversity and divergence metrics are primarily limited to those available through the *parallelDist* R package. This includes 41 distance measures, including common metrics used for microbiome analyses, such as Bray–Curtis and Jaccard distance. *FuncDiv* also supports Jensen–Shannon divergence, as well as weighted and unweighted fast UniFrac distances (Hamady *et al.*, 2010). These UniFrac methods are implemented based on a modified version of the UniFrac function from *phyloseq* (McMurdie and Holmes, 2013). The *beta_div_contrib* function wraps these beta diversity methods and rapidly prepares input matrices of taxonomic abundances per sample, restricted to taxa that encode each function separately, with utilities from the *collapse* and *data.table* R packages.

### 2.5 Resource usage

We evaluated the runtime for representative contributional diversity calculations on a test dataset of 1500 metagenomics samples, with 1962 MAGs and 2012 annotated functions. This dataset was a random subsampling of a larger meta-analysis (Almeida *et al.*, 2019), that was formatted previously to evaluate a different joint taxa-function method (Douglas *et al.*, 2022). On a single core, the elapsed time was 6 h37 m for computing three contributional alpha diversity metrics, and 7 h43 m for computing two contributional beta diversity metrics implemented with the *parallelDist* R package. We also computed weighted UniFrac on this dataset, which took substantial time: 51 h52 m. The maximum memory usage was 2.3, 8.2 and 11.67 GB, respectively, for each respective workflow. All workflow runtimes roughly scaled linearly with the number of input functions and samples (Supplementary Table S1). For instance, the runtimes with 50 and 25% of the starting samples took 3 h33 m and 1 h56 m, respectively, for the alpha diversity workflow. All workflows can be sped up by running multiple cores, e.g. the runtimes were 58 m, 54 m and 6 h 9 m with 10 cores for the alpha, *parallelDist* beta diversity and weighted UniFrac workflows, respectively, on the full dataset.

It is difficult to gauge what the baseline performance for such workflows should be because no other bioinformatic tool computes contributional diversity specifically. One exception is the raw R code provided in the *HUMAnN3* tutorial to compute contributional alpha diversity, which was 60-fold slower than the *FuncDiv* workflow (see Supplementary materials).

### 2.6 Example application

To show how *FuncDiv* results can be used in practice, we computed contributional alpha diversity metrics based on the relative abundances of genera that encode a given MetaCyc pathway within the gut microbiome of individuals tested for soil-transmitted helminths (Rubel *et al.*, 2020). This dataset included 385 pathways encoded by 90 genera in 175 samples (89 soil-transmitted helminth positive and 86 control). We then built Random Forest models to classify samples as helminth positive or control individuals on each input table independently (see Supplementary materials). We also ran models with values for an alpha diversity metric and the genera abundances combined in the input table. On average, the model based on genera abundances performed slightly better than those based on the metrics alone (out-of-bag accuracy of 70% versus a mean of 68%). However, the most informative pathways in these models differed substantially, indicating that they can in principle provide complementary information (Fig. 1b). In addition, the combined models of metric values and genera relative abundances performed best (mean: 74% accuracy), again indicating that the contributional alpha diversity provides complementary information to taxonomic abundances.

## 3 Conclusions

*FuncDiv* enables researchers to investigate the contributional diversity of taxa encoding functions in metagenomic datasets. The computation is fast running and requires low memory usage for standard microbiome datasets. *FuncDiv* provides results that complement standard analyses focused on either taxa or microbial functions alone, enabling new insights into complex datasets.

## Supplementary Material

btac809_Supplementary_Data

## Data Availability

All datafiles and scripts used for the analyses described in our manuscript are available at https://github.com/gavinmdouglas/FuncDiv_manuscript.
