# Supplementary material for: Efficient computation of contributional diversity metrics from microbiome data with FuncDiv
Source: Bioinformatics. 2022 Dec 15;39(1):btac809. doi: 10.1093/bioinformatics/btac809 (PMC9825779; doi:10.1093/bioinformatics/btac809)
Supplement: btac809_Supplementary_Data [file btac809_supplementary_data.pdf]

Supplementary materials for: **Efficient computation of contributional diversity metrics from microbiome data with *FuncDiv***

Comparison with raw R code to compute contributional alpha diversity

As alluded to in the main text, the *HUMAnN3* (Beghini *et al.*, 2021) online tutorial contains the only other workflow that we know of for computing contributional diversity. The example R code provided in this tutorial is not meant to be a general solution for computing contributional diversity, unlike *FuncDiv*, as it is meant to be used specifically with a *HUMAnN3* output table and only computes a single alpha diversity metric (and does not compute beta diversity). Nonetheless, this R code represents a valid approach for computing contributional diversity, and can be compared to help gauge *FuncDiv*'s performance. To do so, we ran the *HUMAnN3* example code to compute the Gini-Simpson index for all pathways in their example output table, which consisted of 78 samples, 254 pathways, and 182 taxa. We also ran *FuncDiv* to compute this metric (on one core) on the same table, and compared the raw elapsed time in seconds for each approach. We ran each approach 10 times to ensure that our estimates were robust. This resulted in mean elapsed times of 21.92s (standard deviation: 1.06s) for the raw tutorial R code and 0.36s (standard deviation: 0.08s) for *FuncDiv*, which represents a 60-fold faster runtime for *FuncDiv*.

Further details on example Random Forest application

Due to space restrictions, several details were left out of our description of the example *FuncDiv* data application (i.e., the Random Forest analysis). The metagenomic dataset we analysed was previously pre-processed as part of the *curatedMetagenomicData* R package (Pasolli *et al.*, 2017). We downloaded the 2021/10/14 version of this processed dataset, which included the relative abundances of taxa across samples, and a breakdown of which genera contribute each pathway per sample. These exact tables in the *curatedMetagenomicData* repository correspond to *2021-10-14.RubelMA_2020.relative_abundance* and *2021-10-14.RubelMA_2020.pathway_abundance* (stratified), respectively. We excluded the *UNMAPPED* and *UNINTEGRATED* rows, as well as rows with unclassified contributors, in the pathway abundance table. We also removed the rows corresponding to the overall pathway abundance

(i.e., the rows containing the sum of abundances across all contributing taxa). We regrouped the taxa relative abundance table to be at the genus level only, to match the contributor levels of the stratified pathway table.

There are numerous approaches for inferring a reconstructed pathway's abundance. For instance, in *HUMAnN3*, the pathway abundances are based on the relative abundances of likely gene family members underlying the pathway. This is also done based on the overall pathway abundance per sample (agnostic to which taxa contribute the gene families). This is a valid approach, but complicates the interpretation of contributional diversity. We instead used the abundances of genera themselves that contribute to each pathway, rather than the reconstructed pathway abundances. In other words, we used the *HUMAnN3* pathway abundance output to identify which genera were contributors of each pathway per sample, and then computed contributional diversity based on the genera relative abundances.

We computed nine alpha contributional metrics per pathway per sample based on this table: Berger Parker's dominance, ENS_pie, Gini-Simpson, Heip's evenness, Inverse Simpson, Pielou's evenness, Richness (observed in data, not estimated), Simpson's evenness, and the Shannon index. We mean-centred and scaled by sample each of these resulting contributional alpha diversity tables.

We then built Random Forests (Breiman, 2001) based on each of these tables, which classified samples as positive or negative for soil-transmitted helminths. We also built a model based on the genera relative abundances alone, with no pathway or contributional diversity information. Prior to building this model, we added a pseudocount of 0.1% to the table of relative abundances, and then performed centred log-ratio transformation by sample. Finally, we built additional Random Forest models with the transformed genera abundances and contributional diversity metrics in the same input table (i.e., a different input table for each diversity metric). The Random Forest models were built with the *ranger* R package v0.14.1 (Wright and Ziegler, 2017) with 10,000 trees and variable importance determined through permutation. The UpSet plot in Figure 1 was created using the *ComplexUpset* R package v1.3.3 (https://krassowski.github.io/complex-upset/index.html).

Resource usage

We selected three alpha diversity metrics for our resource usage assessments: (observed) richness, Gini-Simpson index, and Faith's phylogenetic diversity. For our assessment of the *parallelDist*-based beta diversity metrics, we ran binary (i.e., 1 – Jaccard Similarity) and Bray-Curtis distances. We also ran weighted UniFrac as a separate assessment, which is not implemented through C++ code, and so is much slower compared to *parallelDist*, but nonetheless is one of the key beta diversity metrics used in the microbiome field. We measured resource usage with GNU time v1.7, with the -v option. Runtime and memory usage were parsed from the "Elapsed (wall clock) time" and "Maximum resident set size" lines, respectively, in the output.

Dependency versions

Our reported results are based on *FuncDiv* version 0.4.2. Numerous dependencies are required, which had the following versions: *ape* v5.6.2 (Paradis and Schliep, 2019), *collapse* v1.8.9 (https://sebkrantz.github.io/collapse/index.html), *data.table* v1.14.4, *Rcpp* v1.0.9 (Eddelbuettel and Francois, 2011; Eddelbuettel and Balamuta, 2018), *RcppArmadillo* v0.11.4.0.1 (Eddelbuettel and Sanderson, 2014), *RccpXPtrUtils* v0.1.2 (https://github.com/Enchufa2/RcppXPtrUtils), *parallel* v4.1.1, and *parallelDist* v0.2.6 (https://github.com/alexeckert/parallelDist). These tools are all actively maintained (e.g., all have been updated within 2022), so we do not anticipate any issues relying on these dependencies for *FuncDiv* for the near future. Of course, if incompatibilities do arise with newer versions of R and these dependencies, which we think is unlikely give that they are actively maintained, we would adapt our code appropriately.

References

Beghini,F. *et al.* (2021) Integrating taxonomic, functional, and strain-level profiling of diverse microbial communities with biobakery 3. *eLife*, **10**, e65088.

Breiman,L. (2001) Random Forests. *Mach. Learn.*, **45**, 5–32.

Eddelbuettel,D. and Balamuta,J.J. (2018) Extending R with C++: A Brief Introduction to Rcpp. *Am. Stat.*, **72**, 28–36.

Eddelbuettel,D. and Francois,R. (2011) Seamless R and C++ integration with Rcpp. *Rcpp Seamless R C Integr.*, **40**, 1–18.

Eddelbuettel,D. and Sanderson,C. (2014) RcppArmadillo: Accelerating R with high-performance C++ linear algebra. *Comput. Stat. Data Anal.*, **71**, 1054–1063.

Paradis,E. and Schliep,K. (2019) ape 5.0: An environment for modern phylogenetics and evolutionary analyses in R. *Bioinformatics*, **35**, 526–528.
Pasolli,E. *et al.* (2017) Accessible, curated metagenomic data through ExperimentHub. *Nat. Methods*, **14**, 1023–1024.
Wright,M.N. and Ziegler,A. (2017) ranger: A fast implementation of Random Forests for high dimensional data in C++ and R. *J. Stat. Softw.*, **77**, 1–17.

Supplementary Table 1: Resource usage (elapsed time and maximum memory) to compute representative contributional metrics on datasets of varying complexity

Dataset					***Alpha diversity***[a]		***Beta diversity***[b]		***Weighted UniFrac***	
Description	No. cores	No. samples	No. functions	No. taxa	Time (h:m:s)	Mem. (GB)	Time (h:m:s)	Mem. (GB)	Time (h:m:s)	Mem. (GB)
Complete	10	1500	2012	1962	0:57:34	2.05	0:54:12	8.47	6:08:31	12.55
50% functions	10	1500	1006	1962	0:23:06	1.12	0:39:32	4.37	3:28:01	6.18
50% samples	10	750	2002	1751	0:22:50	1.18	0:15:10	3.63	1:07:25	5.17
25% functions	10	1500	503	1962	0:10:34	0.63	0:17:33	2.73	1:54:28	3.76
25% samples	10	375	1925	1559	0:11:53	0.62	0:01:54	1.74	0:14:00	2.26
Complete	1	1500	2012	1962	6:36:51	2.31	7:42:52	8.20	51:52:29	11.67
50% functions	1	1500	1006	1962	3:28:06	1.14	4:17:54	4.47	28:19:24	6.84
50% samples	1	750	2002	1751	3:32:31	1.18	1:27:33	3.46	9:24:23	5.08
25% functions	1	1500	503	1962	1:42:44	0.62	1:59:15	2.72	13:48:45	4.16
25% samples	1	375	1925	1559	1:55:40	0.60	0:13:58	1.57	2:14:44	2.53

[a]Three alpha diversity metrics: (observed) richness, Gini-Simpson index, and Faith's phylogenetic diversity.

[b]Two beta diversity metrics implemented in *parallelDist*: Jaccard and Bray-Curtis distance.
